# Importance of intercultural paradigm in affirmative clinical practice with LGBTIQ+ people

**DOI:** 10.1192/j.eurpsy.2025.87

**Published:** 2025-08-26

**Authors:** I. Zegura

**Affiliations:** Department for Psychotic Disorders, University Psychiatric Hospital Vrapce, Zagreb, Croatia

## Abstract

**Abstract:**

Professionals’ attitudes, cultural background, and previous experiences are relatively stable and organized features of their backgrounds. They can affect the therapeutic process and provision of health care to LGBTIQ+ clients in both directions- affirmative and oppressive. Healthy development of sexual orientation and gender identity and their integration is one obvious prerequisite for healthy psycho-sexual and psychological development. Mental health care professionals should be aware of their attitudes, and cultural background, and how they can influence the therapeutic process with and provision of health care to LGBTIQ+ clients to provide culturally sensitive and LGBTIQ+ affirmative practice.

LGBTIQ+ affirmative health care can include any single or combination of several social, psychological, behavioral, or medical interventions designed to support and affirm an individual’s sexual orientation and/or gender identity.

Stigma-related stressors experienced by LGBTIQ+ people across these levels can take the form of relational trauma, and thus compound general life stressors that represent an additional risk for different stress-related outcomes including poor mental health and health-risk behaviors.

Expectations of rejection might be particularly frequent experiences among transgender and gender-diverse people. Existing data depict LGBTIQ+ populations to be at a greater risk for poor health outcomes and a plethora of risk behaviors.

Transgender and gender diverse (TGD) people most often perceive available healthcare to be of inadequate quality and potentially unsafe. However, up to now healthcare systems across Europe fail to provide equal access for TGD compared to cisgender people It has been estimated that at least 27% of TGD people in Europe fail to access TGD care. Compared to cisgender people and compared to lesbian, gay, bisexual, or intersex people, TGD people are most affected by not having access to appropriate care, and they are among those reporting the highest rates of experiencing bad health.

Access to the highest standard of LGBTIQ+ affirmative health care, including LGBTIQ+ affirmative approach within psychotherapeutic practice, that respects dignity and right of self-determination, personal, physical, and psychological integrity, autonomy, and wellbeing of LGBTIQ+ people, and that relies on evidence-based practice, clinical guidelines and standards of care should be a priority across European health care systems and psychotherapeutic modalities. Education and sensitization of medical, and mental health professionals, psychotherapists, and other health care professionals on sexuality, sexual orientations, gender identities, and diversities should be implemented as core intercultural competencies through the obligatory curriculum courses of health and mental health professionals.

**Disclosure of Interest:**

None Declared

